# Physician–patient sex dyads and management of dyslipidaemia and hypertension in primary care: findings from two population-based studies

**DOI:** 10.1093/ehjqcco/qcag058

**Published:** 2026-04-16

**Authors:** Shun Yi, Mayssam Nehme, Idris Guessous, Carole Clair, Sophie Moeschler, Marie Méan, Pedro Marques-Vidal

**Affiliations:** Department of Medicine, Internal Medicine, Lausanne University Hospital (CHUV) and University of Lausanne, Rue du Bugnon 46, Lausanne 1011, Switzerland; Faculty of Biology and Medicine, University of Lausanne, Lausanne 1011, Switzerland; Division of Primary Care Medicine, Unit of Population Epidemiology, Geneva University Hospitals, Geneva 1205, Switzerland; Division of Primary Care Medicine, Unit of Population Epidemiology, Geneva University Hospitals, Geneva 1205, Switzerland; Gender and Health Unit, Department of Ambulatory Care, Unisanté, Centre for Primary Care and Public Health and University of Lausanne, Lausanne 1010, Switzerland; Department of Medicine, Internal Medicine, Lausanne University Hospital (CHUV) and University of Lausanne, Rue du Bugnon 46, Lausanne 1011, Switzerland; Department of Medicine, Internal Medicine, Lausanne University Hospital (CHUV) and University of Lausanne, Rue du Bugnon 46, Lausanne 1011, Switzerland; Department of Medicine, Internal Medicine, Lausanne University Hospital (CHUV) and University of Lausanne, Rue du Bugnon 46, Lausanne 1011, Switzerland

**Keywords:** Physician–patient sex dyads, Dyslipidaemia, Hypertension, Primary care, Risk-factor management

## Abstract

**Aims:**

The objective of this study was to examine the associations between physician–patient sex dyads and the prevalence, treatment, and control of dyslipidaemia and hypertension in primary care.

**Methods and results:**

We analysed two Swiss population-based studies using mixed-effects logistic models to compare dyslipidaemia and hypertension management across four physician–patient sex dyads (male physician–male patient, male physician–female patient, female physician–male patient, and female physician–female patient). Age-stratified analyses (≥50, ≥60 years) examined whether dyad–outcome associations differed by patient age. Among 8635 participants (54.9% females, with a mean age of 63 years in the CoLaus study and 48 years in the Bus Santé study), female participants had a lower prevalence of dyslipidaemia and hypertension than male participants across both study samples, irrespective of physician sex. For dyslipidaemia, in CoLaus, female participants had higher odds of lipid-lowering therapy, yet lower odds of low-density lipoprotein cholesterol control, regardless of physician sex; in Bus Santé, female participants managed by male physicians had lower odds of lipid control (adjusted odds ratios (OR) 0.52, 95% confidence intervals (CI) 0.28–0.95) compared to male physician–male patient dyads. For hypertension, in CoLaus, female physician–female patient dyads had lower odds of antihypertensive treatment (adjusted OR 0.62, 95% CI 0.42–0.92) than male physician–male patient dyads; in Bus Santé, treatment and control did not differ significantly by dyad. Most differences were more pronounced in patients aged 60 years or older.

**Conclusion:**

In primary care, patterns of risk-factor management were associated with both patient and physician sex, with dyad-related differences that may reflect patient-sex patterns but differ in magnitude across regions and age groups.

## Graphical Abstract

**Graphical Abstract:**
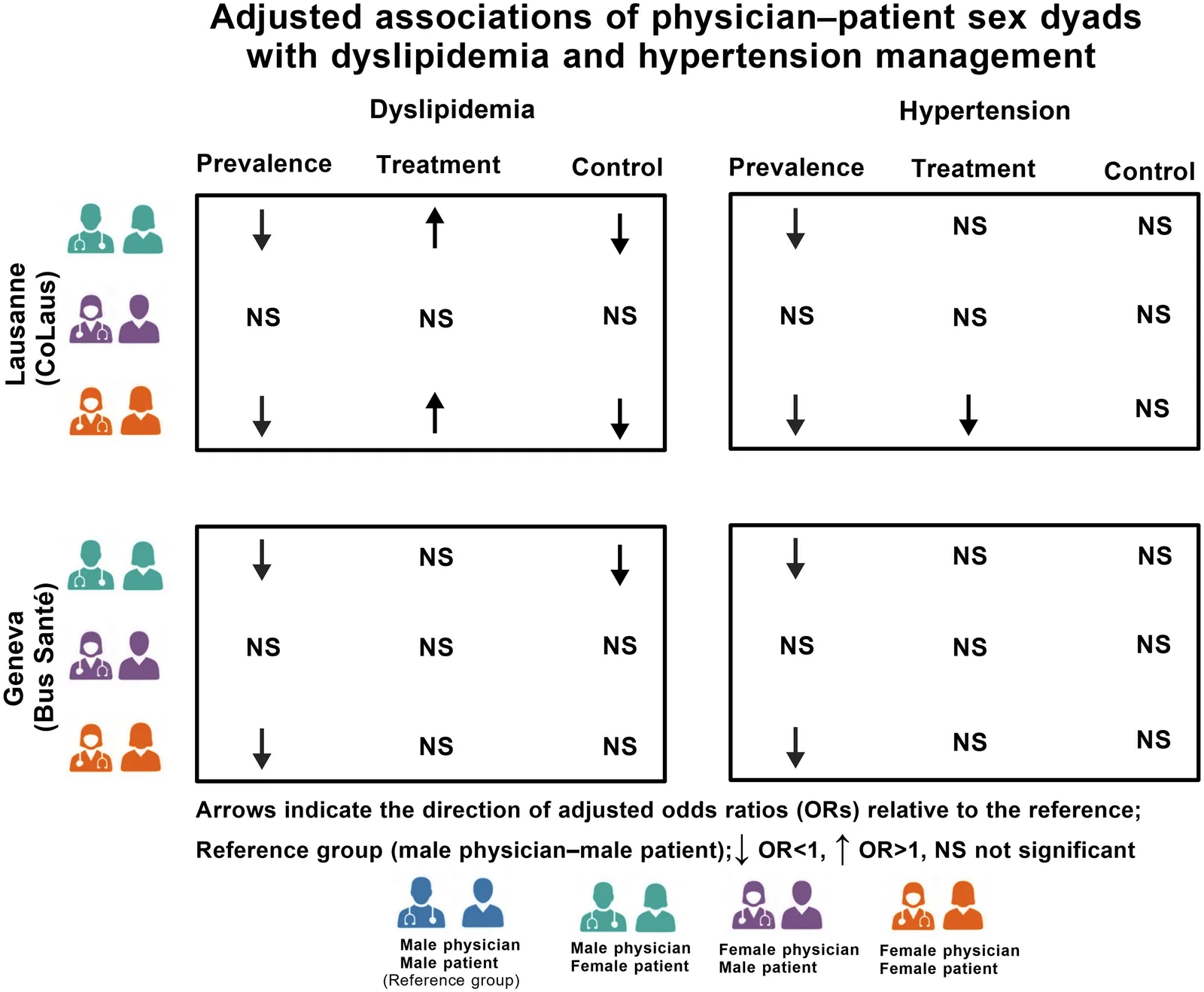

Key Learning Points **What is already known:**

- Sex differences exist in the management of dyslipidaemia and hypertension: females are less likely to receive guideline-recommended statins and achieve low-density lipoprotein cholesterol goals.
- Many previous studies collapse the four dyads into concordant vs. discordant categories, obscuring dyad-specific patterns; contextual factors (e.g. region, age structure) may also shape management but are under-characterized in population-based settings.

**What this study adds:**

- Across two population-based samples (older CoLaus study sample; younger Bus Santé study sample), patient sex, not physician sex, was the primary association of prevalence, treatment, and control; dyad associations were context-dependent.
- In CoLaus study sample, female patients were more frequently treated for dyslipidaemia, yet were less likely to achieve low-density lipoprotein cholesterol control; in Bus Santé study sample, female patients managed by male physicians were less likely to achieve lipid control.
- For hypertension, female physician–female patient dyads had lower odds of antihypertensive treatment in the CoLaus study sample, while blood pressure control did not differ significantly across dyads.

## Introduction

Cardiovascular risk-factor management in primary care, specifically the pharmacologic initiation and control of dyslipidaemia and hypertension, is based on risk-stratified thresholds and targets from the contemporary European guidelines.^1,2^ Despite such benchmarks, sex differences persist along the management cascade: females are less likely to receive guideline-recommended statins and, even when treated, are less likely to attain low-density lipoprotein cholesterol (LDL-C) goals than males.^3,4^ These gaps, not fully explained by biology alone, point to variation in how care is delivered. In particular, for hypertensive patients, male patients are more likely to have fewer risk factors controlled compared to females.^5^ Additionally, delayed initiation of statins has been associated with a higher risk of myocardial infarction.^6^ These findings suggest that sex-based differences in the management and timing of interventions impact outcomes, further emphasizing the importance of considering both physician and patient sex in treatment strategies.

Previous research suggests that female physicians engage in more patient-centred communication and spend more time with patients than male physicians,^7^ which may be associated with differences in treatment decision-making and patient adherence. Beyond communication patterns, differences in clinical decision-making, continuity of care, and patient preferences may also be associated with variation in guideline-concordant prescribing and adherence across the management cascade.^8,9^ Moreover, physician sex, patient sex, and their dyads were associated with cardiovascular risk-factor treatment and control among adults with diabetes.^10^ In routine prevention, female general practitioners were more likely to deliver preventive practices, while female patients managed by male physicians were least likely to receive prevention advice,^11^ highlighting the complex influence of physician–patient sex interactions on preventive care. Similar patterns have been described in a hospital-based process-of-care and outcome study.^12^ Evidence from acute care settings has also reported associations between physician–patient sex dyads and clinically meaningful outcomes, including higher mortality among female patients treated by male physicians in acute myocardial infarction.^13^ A recent systematic review showed that evidence on patient–physician sex concordance remains limited and heterogeneous, largely observational, and derived predominantly from selected clinical populations, with underlying mechanisms incompletely understood.^14^ Similarly, a systematic review suggested potential benefits of female patient–physician concordance for risk-factor management and outcomes; however, evidence across healthcare systems is limited, and the underlying explanations for these associations remain unclear.^15^

However, many studies frequently collapse the four sex dyads into concordant vs. discordant groups,^16,17^ which hides the difference between male physician–female patient and female physician–male patient pairs and makes it hard to separate dyad associations from patient-sex or physician-sex main effects. Less is known about how specific physician–patient sex dyads relate to earlier stages of the management cascade (prevalence, treatment, and control) of major cardiovascular risk factors in routine, population-based primary care, and whether such dyad associations vary across demographic contexts. To clarify these relationships in population-based primary care, analyses should consider all four dyads and account for contextual differences by region and age within the management cascade.

In this study, we assessed whether physician–patient sex dyads are associated with the prevalence, treatment, and control of dyslipidaemia and hypertension in two Swiss population-based study samples. These samples were analysed as complementary settings: CoLaus (an older, longitudinal sample from Lausanne) and Bus Santé (a younger, repeatedly sampled population from Geneva) differ in age structure, population composition, and study design, allowing us to examine whether dyad-related patterns are consistent or context-dependent. We anticipated variation in the magnitude of associations, and analysing both studies strengthens robustness and enables a test of heterogeneity within the same national healthcare system. We further examined heterogeneity by patient age thresholds (≥50 and ≥60 years) to test whether dyad effects depend on age.

## Methods

### Data source and study design

We conducted a cross-sectional analysis based on two population-based studies in Switzerland: CoLaus (2014–2017) and Bus Santé (2014–2019 and 2023–2024; data collection temporarily suspended during the COVID-19 pandemic, 2020–2022). CoLaus investigates the epidemiology and genetic determinants of cardiovascular disease in Lausanne, based on a non-stratified random sample of 19 830 adults aged 35–75 years at baseline.^18^ Bus Santé conducts annual health examination surveys among independent samples of Geneva residents aged 35–74 years, with random selection proportional to age- and sex-specific population frequencies.^19^ Although both study samples are located in the same country, they differ in design and participant composition: CoLaus participants (mean age ≈63 years) were older than those in Bus Santé (mean age ≈48 years), and the educational distributions were inversely structured between the two samples. Given these structural and demographic differences, the two study samples were analysed separately to examine how contextual factors, such as age structure and population composition, may modify associations between physician and patient sex. Key design characteristics of the two study samples are summarized in Supplementary material online, *Table S1*.

### Exposure (physician–patient sex dyads)

The main exposure was the physician–patient sex dyad, categorized into four groups: male physician–male patient (M–M), male physician–female patient (M–F), female physician–male patient (F–M), and female physician–female patient (F–F). The first letter denotes the physician, and the second the patient; M–M served as the reference dyad. Each patient was linked to their general practitioner (GP) using information provided during the study examination. Physician sex (male/female) was retrieved from the Swiss Medical Registry (MedReg), and patient sex was self-reported and recorded as male or female. At the study examination, participants reported a single usual GP. The analytic dataset linked each patient to that physician, and no patient was linked to more than one physician in the analyses.

### Outcomes

Dyslipidaemia was operationalized using study measurements and questionnaire-based medication data, in accordance with the European Society of Cardiology (ESC) 2021 prevention guidance, and defined as eligibility for lipid-lowering therapy (high/very-high cardiovascular risk per guideline criteria) or current use of hypolipidemic medication.^1^ Prevalence of dyslipidaemia was assessed among all study participants. Medication use was assessed among participants meeting the definition of dyslipidaemia (denominator: patients with dyslipidaemia), based on self-reported use of hypolipidemic medication. Control was assessed among treated participants (denominator: patients receiving lipid-lowering therapy) and defined as LDL-cholesterol <2.6 mmol/L, a threshold widely used in population studies assessing lipid control.^20^

Hypertension was defined as systolic blood pressure (SBP) ≥140 mmHg and/or diastolic blood pressure (DBP) ≥90 mmHg, or current antihypertensive medication. The prevalence of hypertension was assessed among all study participants. Medication use was assessed among participants meeting the definition of hypertension (denominator: patients with hypertension), based on self-reported use of antihypertensive medication. Hypertension control was assessed among treated participants (denominator: patients receiving antihypertensive therapy) and defined as SBP <140 mmHg and DBP <90 mmHg in line with the 2023 ESH Guidelines recommendations.^2^

### Covariates

Covariates were selected *a priori* based on clinical relevance and established cardiovascular risk determinants, consistent with prior population-based studies of cardiovascular risk-factor management.^21^ Patient-level covariates were age (years), education (primary/secondary/tertiary), smoking (never/former/current), and body mass index (BMI; kg/m^2^; <24.9, 25.0–29.9, ≥30.0). Physician-level covariates were age (from year of birth to year of data collection), nationality (Swiss/non-Swiss), and years in practice (from medical licensure to year of data collection), retrieved from MedReg.

### Exclusion criteria

We excluded patients with missing physician information or incomplete covariates.

### Statistical analysis

Descriptive analyses for patient and physician characteristics were summarized as means (standard deviations) or numbers (percentages) and compared groups using chi-square tests for categorical variables and either Student’s *t*-tests or Kruskal–Wallis tests for continuous variables, as appropriate.

For the multivariable analysis of each outcome (prevalence, medication use, and control), we fitted mixed-effects logistic regression models with a physician-level random intercept to account for the clustering of patients within physicians. Sex dyads were modelled as a four-level categorical fixed effect (M–M [reference], M–F, F–M, and F–F), allowing separate estimation of each dyad relative to the reference category. Modelling dyads as four distinct categories preserves the differences between concordant and discordant pairs, enabling a more precise estimation of their effects. Models adjusted for patient-level covariates (age, education, smoking, and BMI) and physician-level covariates (age, nationality, and years in practice). We reported adjusted odds ratios (ORs) with 95% confidence intervals (CIs).

Additionally, age-stratified analyses were conducted among patients aged 50 years or older, used as a proxy for post-menopausal status in females.^22^ Because <15% of patients were <50 years, this subgroup was not analysed as a separate stratum. We further conducted age-stratified analyses in patients aged 60 years or older to assess whether associations varied across higher age thresholds, rather than creating multiple narrow age strata due to limited sample sizes.

All analyses were conducted in Stata version 18 (StataCorp, College Station, TX, USA); two-sided tests with *P* < 0.05 were considered statistically significant.

## Ethical approval

The CoLaus study was approved by the institutional Ethics Committee of the University of Lausanne (later the Vaud Cantonal Ethics Commission; project PB_2018-00038, ref 239/09). The Bus Santé study was approved by the Geneva Cantonal Research Ethics Commission (IRB00003116). All studies were performed in accordance with the Helsinki Declaration and its subsequent amendments, and in compliance with applicable Swiss legislation. All patients provided written informed consent.

## Results

### Study sample characteristics

The analyses included 3717 patients from CoLaus and 4918 from Bus Santé (Supplementary material online, *Figure S1*). Baseline characteristics of included and excluded patients are shown in Supplementary material online, *Table S2*. In both study samples, the included patients were broadly similar to those excluded, with some differences. In CoLaus, included patients were less often current smokers and had a lower prevalence of hypertension. In Bus Santé, included patients were older, more often female, less educated, and had a higher prevalence of dyslipidaemia and hypertension.

Among included patients, females were less educated, more often non-smokers, had lower BMI, and had a lower prevalence of dyslipidaemia and hypertension than males in both study samples. Females were slightly older than males in CoLaus but slightly younger in Bus Santé. Among physicians, females were younger and had fewer years in practice than males in both study samples (*Table 1*).

**Table 1:** Baseline characteristics and cardiovascular risk profiles of patients and physicians by sex

	CoLaus			Bus Santé		
Patients	Female	Male	*P*-value	Female	Male	*P*-value
Sample size	2071	1646		2668	2250	
Age (years)	63.2 ± 10.3	62.0 ± 10.1	<0.001	47.0 ± 14.4	48.8 ± 14.5	<0.001
Education level (%)						
Primary	1133 (54.7)	808 (49.1)	<0.001	290 (10.9)	207 (9.2)	0.004
Secondary	572 (27.6)	404 (24.5)		1058 (39.7)	828 (36.8)	
Tertiary	366 (17.7)	434 (26.4)		1320 (49.5)	1215 (54.0)	
Smoking status (%)						
Never	971 (46.9)	586 (35.6)	<0.001	1457 (54.6)	1037 (46.1)	<0.001
Former	732 (35.4)	745 (45.3)		700 (26.2)	723 (32.1)	
Current	368 (17.8)	315 (19.1)		511 (19.2)	490 (21.8)	
BMI category (%)						
Normal	1033 (49.9)	524 (31.8)	<0.001	1753 (65.7)	1002 (44.5)	<0.001
Overweight	677 (32.7)	784 (47.6)		601 (22.5)	920 (40.9)	
Obese	361 (17.4)	338 (20.5)		314 (11.8)	328 (14.6)	
Dyslipidaemia (%)	856 (41.5)	1259 (76.8)	<0.001	659 (24.7)	1174 (52.2)	<0.001
Hypertension (%)	906 (43.8)	908 (55.2)	<0.001	543 (20.4)	635 (28.2)	<0.001

Physicians	Female	Male	*P*-value	Female	Male	*P*-value
Sample size	184	396		359	495	
Age (years)	49.6 ± 9.5	56.1 ± 10.6	<0.001	51.0 ± 9.3	56.4 ± 10.8	<0.001
Practice (years)	22.0 ± 9.6	28.4 ± 11.1	<0.001	22.4 ± 9.2	27.7 ± 10.9	<0.001
Nationality (%)						
Swiss	159 (86.4)	353 (89.1)	0.342	272 (75.8)	416 (84.0)	0.003
Non-Swiss	25 (13.6)	43 (10.9)		87 (24.2)	79 (16.0)	

Data are presented as mean ± standard deviation (SD) for continuous variables and as number (percentage) for categorical variables. Between-group comparisons were performed using the chi-square test for categorical variables, and either Student’s *t*-test or Kruskal–Wallis test for continuous variables, as appropriate. BMI = Body mass index. Years in practice refers to the number of years since medical licensure.

### Unadjusted comparisons by physician–patient sex dyads

Crude comparisons (Supplementary material online, *Table S3*) showed that dyads involving female patients (M–F and F–F) had a lower prevalence of dyslipidaemia and hypertension in both study samples. In CoLaus, treatment among patients with dyslipidaemia was more frequent in dyads with female patients, whereas lipid control was less often achieved in these dyads. For hypertension, the proportion of patients receiving treatment was the highest in F–M dyads and the lowest in F–F dyads, while no differences in blood pressure control were observed across dyads. In Bus Santé, differences in treatment and control were statistically significant only for hypertension.

### Adjusted associations between physician–patient sex dyads and dyslipidaemia

Compared to M–M dyads, dyads involving female patients (M–F and F–F) had a lower prevalence of dyslipidaemia in both study samples. In CoLaus, dyads involving female patients also had higher odds of treatment among those with dyslipidaemia but lower odds of LDL-C control among those treated. In Bus Santé, the M–F dyads had lower odds of LDL-C control (*Table 2*).

**Table 2:** Adjusted associations between physician–patient sex dyads and dyslipidaemia management

Physician sex		Male			Female		
Patient sex	Male	Female		Male		Female	
	Reference	OR (95% CI)	*P*-value	OR (95% CI)	*P*-value	OR (95% CI)	*P*-value
CoLaus							
Sample size (N)	1364	1559		282		512	
Prevalence	1.00 (ref)	0.17 (0.14–0.21)	**<0**.**001**	1.34 (0.94–1.92)	0.103	0.19 (0.14–0.25)	**<0**.**001**
Medication use^a^	1.00 (ref)	1.96 (1.58–2.43)	**<0**.**001**	1.05 (0.75–1.47)	0.788	1.73 (1.23–2.43)	**0**.**002**
Control^b^	1.00 (ref)	0.54 (0.39–0.75)	**<0**.**001**	0.73 (0.42–1.27)	0.270	0.43 (0.26–0.71)	**0**.**001**
Bus Santé							
Sample size (N)	1615	1517		635		1151	
Prevalence	1.00 (ref)	0.28 (0.24–0.34)	**<0**.**001**	0.97 (0.78–1.21)	0.807	0.28 (0.23–0.34)	**<0**.**001**
Medication use^a^	1.00 (ref)	0.97 (0.69–1.35)	0.835	1.38 (0.99–1.92)	0.058	1.12 (0.76–1.66)	0.560
Control^b^	1.00 (ref)	0.52 (0.28–0.95)	**0**.**033**	1.34 (0.76–2.37)	0.318	0.95 (0.47–1.90)	0.876

Odds ratios (ORs) and 95% confidence intervals (CIs) were estimated using mixed-effects logistic regression models with a random intercept for physicians to account for clustering of patients within physicians. All models were adjusted for patient-level covariates (age, education, smoking status, BMI) and physician-level covariates (age, nationality, years in practice). The male patient–male physician dyad served as the reference group. Bold values indicate statistically significant associations (*P* < 0.05). Sample size (N) refers to the number of physician–patient sex dyads within each group (e.g. male patient–male physician).

^a^Medication use was assessed among patients with dyslipidaemia.

^b^Control was assessed among those on medication.

### Adjusted associations between physician–patient sex dyads and hypertension

Compared with M–M dyads, dyads involving female patients (M–F and F–F) had a lower hypertension prevalence in both study samples. In CoLaus, among patients with hypertension, the F–F dyads had lower odds of antihypertensive treatment. In Bus Santé, no significant differences in treatment and control were observed across dyads (*Table 3*).

**Table 3:** Adjusted associations between physician–patient sex dyads and hypertension management

Physician sex		Male			Female		
Patient sex	Male	Female		Male		Female	
	OR (95% CI)	OR (95% CI)	*P*-value	OR (95% CI)	*P*-value	OR (95% CI)	*P*-value
CoLaus							
Sample size (N)	1364	1559		282		512	
Prevalence	1.00 (ref)	0.60 (0.50–0.71)	**<0**.**001**	0.91 (0.67–1.22)	0.522	0.56 (0.44–0.72)	**<0**.**001**
Medication use^a^	1.00 (ref)	1.05 (0.82–1.36)	0.679	1.08 (0.67–1.72)	0.756	0.62 (0.42–0.92)	**0**.**018**
Control^b^	1.00 (ref)	1.22 (0.94–1.58)	0.145	1.11 (0.71–1.75)	0.646	1.03 (0.68–1.57)	0.874
Bus Santé							
Sample size (N)	1615	1517		635		1151	
Prevalence	1.00 (ref)	0.77 (0.64–0.93)	**0**.**007**	1.01 (0.79–1.28)	0.955	0.80 (0.65–0.99)	**0**.**039**
Medication use^a^	1.00 (ref)	0.82 (0.58–1.16)	0.262	1.16 (0.76–1.75)	0.493	0.73 (0.49–1.08)	0.119
Control^b^	1.00 (ref)	1.27 (0.78–2.04)	0.336	0.71 (0.42–1.21)	0.207	1.75 (0.98–3.14)	0.059

Odds ratios (ORs) and 95% confidence intervals (CIs) were estimated using mixed-effects logistic regression models with a random intercept for physicians to account for clustering of patients within physicians. All models were adjusted for patient-level covariates (age, education, smoking status, BMI) and physician-level covariates (age, nationality, years in practice). The male patient–male physician dyad served as the reference group. Bold values indicate statistically significant associations (*P* < 0.05). Sample size (N) refers to the number of physician–patient sex dyads within each group (e.g. male patient–male physician).

^a^Medication use was assessed among patients with hypertension.

^b^Control was assessed among those on medication.

### Age-stratified analyses

Age-stratified analyses by patient age groups (≥50 years and ≥60 years) are presented in Supplementary material online, *Tables S4* and *S5*.

For dyslipidaemia, in CoLaus, dyads involving female patients (M–F and F–F) were associated with lower odds of dyslipidaemia, higher odds of treatment among those with dyslipidaemia, and lower odds of LDL-C control among those treated across both strata; these associations were more pronounced in the ≥60-year group. In Bus Santé, dyads with female patients also had lower odds of dyslipidaemia at both age thresholds; the higher odds of treatment observed for F–M dyads reached significance in the ≥60-year group, while no differences in LDL-C control by dyad were observed.

For hypertension, dyad patterns were broadly similar across age strata: in CoLaus, dyads with female patients (M–F and F–F) had lower odds of prevalent hypertension across age strata and F–F dyads had lower odds of treatment (particularly ≥60 years), whereas control did not differ; in Bus Santé, prevalence was lower in dyads with female patients (M–F, F–F) in the ≥50-year group, with no significant differences in treatment or control at either age strata.

## Discussion

Across two population-based study samples, patterns of risk-factor management varied by both patient and physician sex, with dyad-related differences largely reflecting patient-sex patterns but differing in magnitude across regions and age groups.

### Dyslipidaemia management

Female patients had lower odds of dyslipidaemia than male patients in both study samples. Among those with dyslipidaemia, treatment initiation varied by physician–patient sex dyad. In CoLaus (Lausanne), female patients were nearly twice as likely as male patients to be on lipid-lowering therapy (regardless of physician sex), whereas in Bus Santé (Geneva), no significant sex-dyad differences in treatment were observed. LDL-cholesterol control was also imperfect: in CoLaus, dyads involving female patients (M–F and F–F) were less likely to reach LDL-C targets than M–M dyads, whereas in Bus Santé, M–F dyads had lower odds of reaching LDL-C control. These regional differences may reflect the practice patterns. CoLaus patients were older (mean ∼63 years) than those in Bus Santé (∼48 years), meaning many females in the CoLaus study were post-menopausal and potentially at increased cardiovascular risk.^23^ It may also indicate that physicians in the CoLaus study managed older female patients more proactively with medication. In the Bus Santé study’s younger study sample, the lower LDL-C control observed among M-F dyads may reflect differences in risk perception or treatment intensity, with physicians possibly perceiving younger females as lower risk and thus less in need of intensive lipid-lowering management. We could not assess clinicians’ risk perception or prescribing rationale in this study; hence, this interpretation should be viewed as a hypothesis. Age-stratified analyses showed stronger associations in older participants. In CoLaus, sex disparities in treatment were amplified among older patients: females over 60 were more likely to receive lipid-lowering medication than same-aged males; however, females still showed lower odds of LDL control, which could reflect lower adherence or less intensive therapy among older females. In younger groups, these sex-dyad differences were attenuated, likely because younger females, having fewer risk factors, less often meet thresholds for therapy under risk-based guidelines.^1^ Regarding control, younger females were generally more likely to have a more favourable lipid profile,^24^ which may facilitate LDL-C goal attainment. In Bus Santé, F-M dyads in the older group had higher odds of treatment initiation compared with M-M dyads, whereas this difference was not observed in the younger group, consistent with treatment eligibility increasing with age under risk-based guidelines.

Differences between the two study samples may also be attributed to demographic and contextual factors, such as age distribution, education level, and BMI, which were controlled for in our models. However, these factors could still play an indirect role through unmeasured variables, such as socioeconomic status, health education, and lifestyle factors, which might influence treatment initiation and patient adherence. These unmeasured factors could potentially contribute to residual confounding and explain some of the observed differences in prevalence rates between the two groups. For instance, educational differences in Bus Santé might reflect variation in engagement with preventive health practices, which could influence treatment receipt and goal attainment. Additionally, there may be a self-selection factor in the physician–patient dyads, as female physicians may attract more female patients. Such self-selection could potentially influence the likelihood of receiving treatment or achieving treatment goals. Dyslipidaemia management is also influenced by more complex guidelines that account for a broader range of risk factors, including comorbidities like diabetes and age.^1^ These guidelines require more nuanced and complex clinical decision-making. These factors may help explain the heterogeneity observed across the two samples, particularly for dyslipidaemia management.

In addition to adherence and treatment intensity, biological differences between males and females may influence the pharmacokinetics and pharmacodynamics of lipid-lowering therapy. Sex differences in drug absorption, distribution, metabolism, and excretion have been reported,^25^ which could affect response to statins. Specifically, females may experience differences in statin tolerability, which could contribute to suboptimal LDL-C control despite adequate treatment. This is consistent with our findings in the CoLaus cohort, where female patients were more frequently treated with lipid-lowering therapy but had lower odds of LDL-C control compared to male patients. However, current evidence on sex-specific differences in LDL-C reduction with statin therapy is limited. While an observational study showed disparities in therapeutic goal attainment,^4^ whether this reflects true biological differences in pharmacological responsiveness or other factors (e.g. dosing, adherence, and side effects) remains unclear, highlighting the need for more sex-specific research in this area.

Our findings both reflect and extend known sex disparities in lipid management. One study reported that female patients are less likely than male patients to receive guideline-recommended statin therapy.^3^ This gap has been attributed not only to clinicians prescribing statins less frequently for females but also to females more often declining or discontinuing therapy.^3^ In this context, the higher medication use observed among female patients in the CoLaus study is likely partly explained by the older age of this sample, where many females were post-menopausal and at higher cardiovascular risk. By contrast, in the younger Bus Santé study, medication use did not differ significantly across physician–patient sex dyads, suggesting that differences in age and cardiovascular risk profile may largely drive the pattern observed in CoLaus. Despite higher odds of treatment, female patients did not achieve LDL-C targets in the CoLaus study. This mirrors the EUROASPIRE V registry, where only 22% of female patients achieved the LDL goal, vs. 31% of males.^4^ Our results show that this gap persists even in primary care and is not fully explained by undertreatment of females, since in our data, female patients in the CoLaus study were more often treated than males. Possible contributors include a higher proportion of statin intolerance or poorer adherence among female patients,^3,26^ underscoring the need to not only initiate therapy in females but also ensure it is sustained and sufficiently intensive to reach goals.

Taken together, findings for dyslipidaemia management showed greater variability, particularly for medication use and control. The age structure differed between the two samples, with CoLaus comprising an older population in whom lipid management is more strongly driven by absolute cardiovascular risk. Differences in population composition may therefore partly explain the greater heterogeneity observed for dyslipidaemia outcomes.

#### Hypertension management

Female patients had a lower prevalence of hypertension than males across both study samples, regardless of physician sex. In the CoLaus study, among patients with hypertension, F–F dyads had lower odds of medication use compared to M–M dyads. The lower medication use in F–F dyads was most pronounced among older females. In the Bus Santé study, no significant sex-dyad differences in treatment were observed. One possible explanation is that CoLaus included an older population, in which polypharmacy is more common among older females than males,^27^ potentially encouraging more cautious pharmacologic initiation despite eligibility. In the Bus Santé study, no significant sex-dyad differences in treatment were observed, possibly because this younger population included many females with milder hypertension or lower baseline blood pressure,^28^ making lifestyle-first management a more common and guideline-consistent approach.^2^ Once patients were on antihypertensive therapy, we did not observe sex-based differences in blood pressure control across study samples. Male and female physicians achieved comparable control in their patients, and no significant differences were found across physician–patient sex dyads. This consistency across study samples may indicate that blood pressure management, once pharmacologic therapy is initiated, may be less influenced by sex-related factors and more by standardized clinical protocols and follow-up practices in primary care. However, we could not assess follow-up intensity or adherence in this study.

Previous research on sex differences in hypertension treatment has yielded mixed results. A study reported that females were less likely to receive antihypertensive drug treatment than males,^29^ which is consistent with our finding of lower medication use in F–F dyads in the CoLaus study. However, other studies found no significant sex differences in treatment or even higher antihypertensive use among females.^30,31^ These discrepancies may reflect differences in study populations and healthcare systems. Our results raise the possibility that the physician’s sex may influence management patterns. In particular, lower odds of treatment observed when female patients were managed by female physicians may reflect a unique interaction between patient and physician decision-making styles, highlighting the importance of considering both patient and physician sex when addressing disparities in care.

### Strengths and limitations

Strengths of this study include the use of two large, well-characterized population-based samples from different regions, which provide insights into primary care patterns in real-world settings. We had detailed information on patients and their physicians, allowing us to adjust for important confounders. We also conducted stratified analyses by age, which confirmed the robustness of our main findings.

Despite these strengths, several limitations should be acknowledged. First, the study design is observational and cross-sectional, which limits the ability to establish causality for the outcomes of interest. Second, exclusion of participants with missing physician information or covariates may have introduced selection bias. Third, physician–patient linkage relied on the GP reported by patients at the time of the study examination. However, the reported GP may not always be the clinician primarily responsible for long-term lipid- or blood-pressure–lowering therapy (e.g. specialist-initiated prescriptions). We also lacked information on GP-driven medication adjustments or discontinuations (including considerations around guideline intensity and polypharmacy), as well as treatment intensity and adherence. Residual confounding due to unmeasured clinical and practice-level factors (e.g. comorbidities, medication contraindications, and consultation frequency) cannot be excluded. These limitations may lead to non-differential misclassification of the physician–patient sex dyad, which would be expected to attenuate dyad-specific associations toward the null. Medication use was self-reported and may be subject to recall bias or misclassification of treatment status. In addition, guideline thresholds for lipid management have evolved over the study periods, spanning multiple ESC guideline updates. To ensure comparability across study samples and calendar years, we retrospectively applied contemporary guideline-based thresholds. This approach may have resulted in minor outcome misclassification in lipid control for some patients. However, such misclassification is unlikely to be systematic and therefore unlikely to alter the overall pattern of associations observed. Although the study included two regions, both are from the same country. Hence, our findings likely reflect primary care dynamics in similar high-income healthcare systems, but their generalization to other settings should be interpreted with caution. The two datasets also cover different time periods, with the CoLaus study spanning 2014–2017 and the Bus Santé study covering 2014–2019, with a gap in data collection from 2020–2022 due to the COVID-19 pandemic. These temporal factors may have influenced care patterns and outcomes and should be considered when interpreting the results. Replication in more diverse healthcare settings would help validate the observed patterns and assess their applicability beyond the Swiss healthcare context. Future studies incorporating more direct or objective measures of medication adherence (e.g. electronic medication monitoring devices) and physician-assessed adherence may help disentangle pharmacological treatment effects from behavioural influences on LDL-cholesterol control.

### Implications for practice

First, differences between regions (Lausanne vs. Geneva) may reflect underlying demographic and risk profile variations, such as age distributions, as well as differences in clinical practice. Interventions may therefore need to account for both regional population characteristics and local care practices. Second, the lower prevalence of dyslipidaemia and hypertension in females should not obscure management gaps (e.g. lower LDL-C goal attainment despite higher treatment in Lausanne). Third, population-level cardiovascular risk-factor surveillance should report sex-stratified indicators, such as prevalence, medication use, and LDL-C control.

## Conclusion

In two large, population-based samples, cardiovascular risk-factor management was mainly associated with patient sex, while physician–patient sex dyads had only context-dependent effects. These findings underscore the need for sex-sensitive strategies in primary care to ensure both equitable treatment initiation and effective risk-factor control. Incorporating sex-specific monitoring and communication training into preventive care may help close management gaps.

## Supplementary Material

qcag058_Supplementary_Data

## Data Availability

The data of the CoLaus|PsyCoLaus study used in this article cannot be fully shared as they contain potentially sensitive personal information on patients. According to the Ethics Committee for Research of the Canton of Vaud, sharing these data would be a violation of the Swiss legislation with respect to privacy protection. However, coded individual-level data that do not allow researchers to identify patients are available upon request to researchers who meet the criteria for data sharing of the CoLaus|PsyCoLaus Datacenter (CHUV, Lausanne, Switzerland). Any researcher affiliated with a public or private research institution who complies with the CoLaus|PsyCoLaus standards can submit a research application to research.colaus@chuv.ch or research.psycolaus@chuv.ch. Proposals will be evaluated by the Scientific Committee (SC) of the CoLaus|PsyCoLaus study. Detailed instructions for gaining access to the CoLaus|PsyCoLaus data used in this study are available at www.colaus-psycolaus.ch/professionals/how-to-collaborate/. Coded data for the Bus Santé study are available upon reasonable request.
