# Supplementary material for: Physician–patient sex dyads and management of dyslipidaemia and hypertension in primary care: findings from two population-based studies
Source: Eur Heart J Qual Care Clin Outcomes. 2026 Apr 16;12(6):1099–107. doi: 10.1093/ehjqcco/qcag058 (PMC13579329; doi:10.1093/ehjqcco/qcag058)
Supplement: Supplementary file 1 [file qcag058_supplementary_data.zip › 20260325_6_Supplementary tables.docx]

### Supplementary information

**Supplementary Figure 1**. **Flowchart of patient inclusion and exclusion**.

# Supplementary Table 1. Key design characteristics of CoLaus and Bus Santé.

|  | **CoLaus** | **Bus Santé** |
| --- | --- | --- |
| **Study design** | Longitudinal population-based cohort | Repeated cross-sectional community-based survey with independent annual samples |
| **Geographic setting** | Lausanne, Canton of Vaud | Geneva, Canton of Geneva |
| **Sampling strategy** | Non-stratified random sampling from the population registry | Age- and sex-stratified random sampling proportional to population distribution |
| **Age range** | 35–75 years at baseline; older mean age in analytic sample (mean age ≈63 years) | 35–74 years; younger mean age in analytic sample (mean age ≈48 years) |
| **Data collection** | Standardised questionnaire and clinical examination | Standardised questionnaire and clinical examination |
| **Blood pressure measurement** | Measured during study visit using standardised protocol | Measured during study visit using standardised protocol |
| **Lipid assessment** | Blood sampling during examination with laboratory analysis | Blood sampling during examination with laboratory analysis |
| **GP identification** | Participant-reported GP, linked to national medical registry (MedReg) | Participant-reported GP, linked to national medical registry (MedReg) |

# Supplementary Table 2. Baseline characteristics and cardiovascular risk profiles of participants included and excluded.

|  | **CoLaus** |  |  | **Bus** | **Santé** |  |
| --- | --- | --- | --- | --- | --- | --- |
| **Characteristic** | **Included** | **Excluded** | **P value** | **Included** | **Excluded** | **P value** |
| **Age (years)** | 62.7±10.2 | 63.7±11.1 | 0.004 | 47.8±14.5 | 43.4±13.6 | <0.001 |
| **Female sex (%)** | 2,071 (55.7) | 618 (53.1) | 0.116 | 2,668 (54.3) | 1,461 (47.5) | <0.001 |
| **Education level (%)** |  |  |  |  |  |  |
| Primary | 1,941 (52.2) | 647 (55.8) | 0.106 | 497 (10.1) | 300 (9.9) | <0.001 |
| Secondary | 976 (26.3) | 282 (24.3) |  | 1,886 (38.3) | 976 (32.3) |  |
| Tertiary | 800 (21.5) | 231 (19.9) |  | 2,535 (51.6) | 1,750 (57.8) |  |
| **Smoking status (%)** |  |  |  |  |  |  |
| Never | 1,557 (41.9) | 329 (42.3) | 0.023 | 2,494 (50.7) | 1,585 (52.7) | <0.001 |
| Former | 1,477 (39.7) | 277 (35.6) |  | 1,423 (28.9) | 735 (24.5) |  |
| Current | 683 (18.4) | 172 (22.1) |  | 1,001 (20.4) | 684 (22.8) |  |
| **BMI category (%)** |  |  |  |  |  |  |
| Normal | 1,557 (41.9) | 300 (39.3) | 0.408 | 2,755 (56.0) | 1,866 (60.7) | <0.001 |
| Overweight | 1,461 (39.3) | 314 (41.1) |  | 1,521 (30.9) | 877 (28.5) |  |
| Obese | 699 (18.8) | 150 (19.6) |  | 642 (13.1) | 330 (10.8) |  |
| **Dyslipidaemia (%)** | 2,115 (57.1) | 577 (59.7) | 0.155 | 1,833 (37.3) | 946 (30.8) | <0.001 |
| **Hypertension (%)** | 1,814 (48.9) | 580 (58.9) | <0.001 | 1,178 (24.0) | 533 (17.3) | <0.001 |

Data are presented as mean ± standard deviation for continuous variables and number (percentage) for categorical variables. Between-group comparisons were performed using the chi-square test for categorical variables and either the student’s t-test or the Kruskal–Wallis test for continuous variables, as appropriate. BMI, Body Mass Index.

**Supplementary Table 3.** Unadjusted comparisons of cardiovascular risk factor management according to physician–patient sex dyads.

| **CoLaus** |  |  |  |  |  |
| --- | --- | --- | --- | --- | --- |
| **Physician Sex** | **Male** | **Male** | **Female** | **Female** | **P value** |
| **Patient Sex** | **Male** | **Female** | **Male** | **Female** |  |
| **Sample size (N)** | 1,364 | 1,559 | 282 | 512 |  |
| **Dyslipidaemia** |  |  |  |  |  |
| Prevalence | 1,037 (76.4) | 645 (41.6) | 222 (79.0) | 211 (41.2) | <0.001 |
| Medication use^1^ | 366 (35.3) | 337 (52.3) | 84 (37.8) | 109 (51.7) | <0.001 |
| Control^2^ | 204 (57.3) | 142 (42.5) | 39 (49.4) | 44 (40.7) | <0.001 |
| **Hypertension** |  |  |  |  |  |
| Prevalence | 760 (55.8) | 696 (44.7) | 148 (52.5) | 210 (41.0) | <0.001 |
| Medication use^1^ | 535 (70.4) | 507 (72.8) | 111 (75.0) | 132 (62.9) | 0.027 |
| Control^2^ | 323 (60.4) | 318 (62.7) | 71 (64.0) | 81 (61.4) | 0.833 |
| **Bus Santé** |  |  |  |  |  |
| **Physician Sex** | **Male** | **Male** | **Female** | **Female** | **P value** |
| **Patient Sex** | **Male** | **Female** | **Male** | **Female** |  |
| **Sample size (N)** | 1,615 | 1,517 | 635 | 1,151 |  |
| **Dyslipidaemia** |  |  |  |  |  |
| Prevalence | 854 (52.9) | 389 (25.6) | 320 (50.4) | 270 (23.5) | <0.001 |
| Medication use^1^ | 183 (21.4) | 69 (17.7) | 79 (24.7) | 49 (18.2) | 0.089 |
| Control^2^ | 95 (51.9) | 27 (39.1) | 46 (58.2) | 26 (53.1) | 0.128 |
| **Hypertension** |  |  |  |  |  |
| Prevalence | 458 (28.4) | 308 (20.3) | 177 (27.9) | 235 (20.4) | <0.001 |
| Medication use^1^ | 230 (50.2) | 135 (43.8) | 91 (51.4) | 92 (39.2) | 0.017 |
| Control^2^ | 140 (60.9) | 91 (67.4) | 51 (56.0) | 68 (73.9) | 0.045 |

Results are presented as numbers (percentages) for categorical variables. Unadjusted between-group comparisons were performed using two-sided chi-square tests. Dyads are defined as male physician–male patient, male physician–female patient, female physician–male patient, and female physician–female patient. Medication use¹ was assessed among patients with dyslipidaemia or hypertension, and Control² among those on medication.

**Supplementary Table 4.** Adjusted associations between physician–patient sex dyads and dyslipidaemia management outcomes, stratified by patient age group.

| **Physician sex** |  | **Male** |  |  | **Female** |  |  |
| --- | --- | --- | --- | --- | --- | --- | --- |
| **Patient sex** | **Male** | **Female** |  | **Male** |  | **Female** |  |
|  | **Reference** | **OR (95% CI)** | **P-value** | **OR (95% CI)** | **P-value** | **OR (95% CI)** | **P-value** |
| **CoLaus** |  |  |  |  |  |  |  |
| **Patients aged ≥50 years** |  |  |  |  |  |  |  |
| Sample size (N) | 1,190 | 1,384 |  | 237 |  | 451 |  |
| Prevalence | 1.00 (ref) | 0.16 (0.13 - 0.20) | **<0.001** | 1.28 (0.86 - 1.92) | 0.226 | 0.18 (0.13 - 0.23) | **<0.001** |
| Medication use^1^ | 1.00 (ref) | 1.86 (1.49 - 2.32) | **<0.001** | 1.09 (0.77 - 1.54) | 0.630 | 1.76 (1.24 - 2.49) | **0.002** |
| Control^2^ | 1.00 (ref) | 0.53 (0.37 - 0.75) | **<0.001** | 0.77 (0.44 - 1.36) | 0.368 | 0.42 (0.25 - 0.70) | **0.001** |
| **Patients aged ≥60 years** |  |  |  |  |  |  |  |
| Sample size (N) | 717 | 922 |  | 133 |  | 274 |  |
| Prevalence | 1.00 (ref) | 0.05 (0.03 - 0.07) | **<0.001** | 1.27 (0.48 - 3.39) | 0.628 | 0.06 (0.04 - 0.09) | **<0.001** |
| Medication use^1^ | 1.00 (ref) | 2.26 (1.73 - 2.94) | **<0.001** | 1.08 (0.70 - 1.68) | 0.716 | 2.24 (1.47 - 3.42) | **<0.001** |
| Control^2^ | 1.00 (ref) | 0.41 (0.28 - 0.60) | **<0.001** | 0.90 (0.46 - 1.75) | 0.758 | 0.35 (0.20 - 0.60) | **<0.001** |
| **Bus Santé** |  |  |  |  |  |  |  |
| **Patients aged ≥50 years** |  |  |  |  |  |  |  |
| Sample size (N) | 791 | 654 |  | 293 |  | 489 |  |
| Prevalence | 1.00 (ref) | 0.22 (0.17 - 0.28) | **<0.001** | 0.93 (0.67 - 1.30) | 0.680 | 0.22 (0.16 - 0.29) | **<0.001** |
| Medication use^1^ | 1.00 (ref) | 0.93 (0.65 - 1.32) | 0.669 | 1.39 (0.97 - 1.98) | 0.070 | 1.11 (0.74 - 1.66) | 0.624 |
| Control^2^ | 1.00 (ref) | 0.56 (0.29 - 1.06) | 0.074 | 1.46 (0.80 - 2.66) | 0.218 | 0.98 (0.47 - 2.03) | 0.959 |
| **Patients aged ≥60 years** |  |  |  |  |  |  |  |
| Sample size (N) | 422 | 340 |  | 152 |  | 223 |  |
| Prevalence | 1.00 (ref) | 0.09 (0.06 - 0.13) | **<0.001** | 1.18 (0.62 - 2.25) | 0.611 | 0.10 (0.06 - 0.15) | **<0.001** |
| Medication use^1^ | 1.00 (ref) | 1.22 (0.79 - 1.88) | 0.378 | 1.58 (1.02 - 2.44) | **0.040** | 1.25 (0.74 - 2.11) | 0.407 |
| Control^2^ | 1.00 (ref) | 0.42 (0.16 - 1.12) | 0.083 | 1.64 (0.63 - 4.24) | 0.308 | 0.70 (0.22 - 2.26) | 0.548 |

Odds ratios (ORs) and 95% confidence intervals (CIs) were estimated using mixed-effects logistic regression models with a random intercept for physicians to account for clustering of patients within physicians. All models were adjusted for patient-level covariates (age, education, smoking status, BMI) and physician-level covariates (age, nationality, years in practice). The male patient–male physician dyad served as the reference group. Bold values indicate statistically significant associations (P < 0.05). Sample size (N) refers to the number of physician–patient sex dyads within each group (e.g., male patient–male physician). Medication use^1^ was assessed among patients with dyslipidaemia, and Control^2^ was assessed among those on medication.

**Supplementary Table 5.** Adjusted associations between physician–patient sex dyads and hypertension management outcomes, stratified by patient age group.

| **Physician sex** |  | **Male** |  |  | **Female** |  |  |
| --- | --- | --- | --- | --- | --- | --- | --- |
| **Patient sex** | **Male** | **Female** |  | **Male** |  | **Female** |  |
|  | **OR (95% CI)** | **OR (95% CI)** | **P-value** | **OR (95% CI)** | **P-value** | **OR (95% CI)** | **P-value** |
| **CoLaus** |  |  |  |  |  |  |  |
| **Patients aged ≥50 years** |  |  |  |  |  |  |  |
| Sample size (N) | 1,190 | 1,384 |  | 237 |  | 451 |  |
| Prevalence | 1.00 (ref) | 0.58 (0.49 - 0.70) | **<0.001** | 0.93 (0.67 - 1.29) | 0.668 | 0.55 (0.42 - 0.71) | **<0.001** |
| Medication use^1^ | 1.00 (ref) | 1.02 (0.79 - 1.33) | 0.872 | 1.02 (0.63 - 1.67) | 0.929 | 0.59 (0.40 - 0.89) | **0.011** |
| Control^2^ | 1.00 (ref) | 1.20 (0.92 - 1.57) | 0.181 | 1.16 (0.72 - 1.85) | 0.544 | 1.03 (0.67 - 1.58) | 0.904 |
| **Patients aged ≥60 years** |  |  |  |  |  |  |  |
| Sample size (N) | 717 | 922 |  | 133 |  | 274 |  |
| Prevalence | 1.00 (ref) | 0.63 (0.50 - 0.79) | **<0.001** | 1.24 (0.79 - 1.96) | 0.348 | 0.48 (0.35 - 0.66) | **<0.001** |
| Medication use^1^ | 1.00 (ref) | 0.88 (0.64 - 1.21) | 0.438 | 1.06 (0.56 - 2.02) | 0.857 | 0.42 (0.26 - 0.70) | **0.001** |
| Control^2^ | 1.00 (ref) | 1.15 (0.86 - 1.55) | 0.351 | 1.04 (0.62 - 1.75) | 0.882 | 0.93 (0.57 - 1.53) | 0.789 |
| **Bus Santé** |  |  |  |  |  |  |  |
| **Patients aged ≥50 years** |  |  |  |  |  |  |  |
| Sample size (N) | 791 | 654 |  | 293 |  | 489 |  |
| Prevalence | 1.00 (ref) | 0.74 (0.58 - 0.93) | **0.011** | 1.08 (0.81 - 1.44) | 0.604 | 0.77 (0.60 – 1.00) | 0.054 |
| Medication use^1^ | 1.00 (ref) | 0.77 (0.52 - 1.13) | 0.180 | 1.02 (0.64 - 1.62) | 0.926 | 0.71 (0.46 - 1.10) | 0.125 |
| Control^2^ | 1.00 (ref) | 1.22 (0.71 - 2.08) | 0.474 | 0.59 (0.32 - 1.07) | 0.084 | 1.62 (0.85 - 3.1) | 0.141 |
| **Patients aged ≥60 years** |  |  |  |  |  |  |  |
| Sample size (N) | 422 | 340 |  | 152 |  | 223 |  |
| Prevalence | 1.00 (ref) | 0.77 (0.57 - 1.05) | 0.101 | 1.14 (0.77 - 1.69) | 0.501 | 0.70 (0.49 - 1.01) | 0.056 |
| Medication use^1^ | 1.00 (ref) | 0.79 (0.47 - 1.33) | 0.373 | 1.31 (0.70 - 2.45) | 0.391 | 0.85 (0.46 - 1.59) | 0.613 |
| Control^2^ | 1.00 (ref) | 0.91 (0.47 - 1.76) | 0.781 | 0.56 (0.26 - 1.19) | 0.130 | 1.65 (0.73 - 3.72) | 0.225 |

Odds ratios (ORs) and 95% confidence intervals (CIs) were estimated using mixed-effects logistic regression models with a random intercept for physicians to account for clustering of patients within physicians. All models were adjusted for patient-level covariates (age, education, smoking status, BMI) and physician-level covariates (age, nationality, years in practice). The male patient–male physician dyad served as the reference group. Bold values indicate statistically significant associations (P < 0.05). Sample size (N) refers to the number of physician–patient sex dyads within each group (e.g., male patient–male physician). Medication use^1^ was assessed among patients with hypertension, and Control^2^ was assessed among those on medication.
